# Development of a New Collagen Gel Product for Leather Finishing

**DOI:** 10.3390/gels9110883

**Published:** 2023-11-08

**Authors:** Xinping Zhang, Sílvia Sorolla, Concepció Casas, Anna Bacardit

**Affiliations:** A3 Leather Innovation Center, Escola Politècnica Superior, Departament d’Informàtica i Enginyeria Industrial, Universitat de Lleida (UdL), 25003 Lleida, Spain; rory@prosper.com.cn (X.Z.); silvia.sorolla@udl.cat (S.S.); concepcio.casas@udl.cat (C.C.)

**Keywords:** biobased products, biodegradable materials, trypsin

## Abstract

Leather finishing is a critical process in the leather industry, as it significantly influences the final appearance, durability, and quality of leather products. Traditional leather finishing techniques often involve the use of synthetic chemicals, which may lead to environmental concerns and potential health hazards. In this study, we investigate the feasibility and effectiveness of a new collagen-based product for leather finishing. Collagen, a natural protein found abundantly in animals, has shown promise as an environmentally friendly and sustainable alternative for leather finishing. The new collagen gel product obtained from bovine hide waste by using an alkaline extraction method with lime was functionalized through an enzymatic treatment that allows to achieve a finishing product suitable for coating formulations, and at the same time, a biodegradable finishing. The collagen gel product was optimized by varying parameters, such as temperature, pH, and enzyme quantity. The optimized collagen gel product exhibits a wide particle size range and retains the triple-helical structure of collagen. The leather samples treated with the collagen gel product show enhanced properties compared to those with conventional finishes. The results show that the collagen gel product enhances water vapor permeability, color stability, and touch in the finishes. However, a low resistance to wet rubbing is obtained; therefore, it is necessary to study how to improve this parameter.

## 1. Introduction

The leather industry is continually striving to develop sustainable and environmentally friendly methods for leather production and finishing. Collagen, a protein responsible for the structural integrity of skin, bones, and connective tissues, has attracted attention as a potential ecofriendly alternative to synthetic finishing agents.

Collagen is the main structural protein in the extracellular matrix found in the body’s various connective tissues. As the main component of connective tissue, it is the most abundant protein in mammals [1], making up from 25% to 35% of the whole-body protein content. Collagen consists of amino acids bound together to form a triple helix of elongated fibril [2] known as a collagen helix. It is mostly found in connective tissue, such as cartilage, bones, tendons, ligaments, and skin.

Depending upon the degree of mineralization, collagen tissues may be rigid (bone) or compliant (tendon) or have a gradient from rigid to compliant (cartilage). Collagen is also abundant in corneas, blood vessels, guts, intervertebral discs, and dentin in teeth. In muscle tissue, it serves as a major component of the endomysium. Collagen constitutes 1 to 2% of muscle tissue and accounts for 6% of the weight of the skeletal muscle tissue [3]. The fibroblast is the most common cell that creates collagen. Gelatine, which is used in the food industry, is collagen that has been irreversibly hydrolyzed by heating, basic solutions or weak acids [4].

Collagen is the most abundant protein in bones and connective tissue in vertebrates, and there are at least 29 types. They are different in terms of their amino acid sequence and composition, function in the organism, and structure [5,6]. The structure of collagen is a triple helix formed for 3-chain Gly–X–Y, where X is proline, Y is mainly hydroxyproline, and the triple helix is stabilized for hydrogen bonds with continuous repetition of the Gly–X–Y depending on the collagen type [7]. Collagen can be extracted from the skin, bones, tendons, and cartilage of pigs [8], cows [9], marine organisms [10,11,12], and rabbits [13]. Hydrolyzed collagen refers to a group of peptides that result from the proteolysis of native collagen type 1; its molecular weight (Mw) varies from 0.3 to 8000 Da [14]. It does not jellify in solution at room temperature and is soluble in cold water, so it can mix easily with other products [15,16,17]. Additionally, hydrolyzed collagen has a neutral smell, is colorless, and can be used in emulsions as a stabilizer. It is widely used in the pharmaceutical industry for the treatment of diseases like osteoarthritis and osteoporosis. Also, in the cosmetic and food industries, it is applied for the preparation of fruity beverages and nutritional supplements [18,19,20,21].

This study aims to explore the use of a collagen-based product for leather finishing and assess its suitability as a replacement for conventional chemical finishes to obtain a biodegradable leather, because collagen, as a fundamental protein in the animal kingdom, exhibits excellent biodegradability. Its organic composition allows for enzymatic breakdown by microorganisms, resulting in the recycling of its constituents in the environment.

Collagen can be broken down into its basic amino acids, including glycine, proline, and hydroxyproline, which are readily degraded by microorganisms in the environment. These microorganisms, such as bacteria and fungi, produce enzymes called collagenases that specifically target collagen and initiate the breakdown process [22].

Collagen degradation occurs through a complex enzymatic process. Initially, collagenases cleave the protein chains into smaller fragments known as peptides. Subsequently, other enzymes, including gelatinases and peptidases, further break down the peptides into individual amino acids. These amino acids can then be assimilated by microorganisms and other organisms, participating in the natural nutrient cycle [23].

This collagen degradation can occur through different pathways, depending on the environmental conditions and the specific microorganisms involved. Aerobic degradation takes place in the presence of oxygen, where bacteria and fungi utilize collagen as a carbon and energy source, converting it into carbon dioxide (CO_2_) and water (H_2_O) through metabolic processes [24].

Fan et al. studied the engineering of nanocomposites by integrating nanoparticles with polymers. Different methods including sol–gel processing, blending, and in situ methods were developed. These methods have applications in developing functional composite leather finishes [25].

As biopolymers offer an excellent avenue for creating and utilizing innovative functional materials, thanks to their unique advantages, such as biocompatibility, biodegradability, and nontoxicity [26], a previous study introduced an innovative finishing formulation that not only embraces ecofriendly technology but also aligns with a circular production approach, utilizing recovered solid wastes from leather. The devised finishing system revolves around applying collagen, extracted from tanned wastes through enzymatic treatment, to cross-link and adhere to the leather surface. The resulting biobased finished leather underwent a comparison with a resin-based finished leather, demonstrating equivalent quality standards as demanded by the market [27].

In a further step, in the present work, the best system for optimizing both the production of a new collagen-based product and its application on leather to achieve a biodegradable finish is being studied.

## 2. Results and Discussion

### 2.1. Collagen Gel Product Obtainment

**Figure 1:**
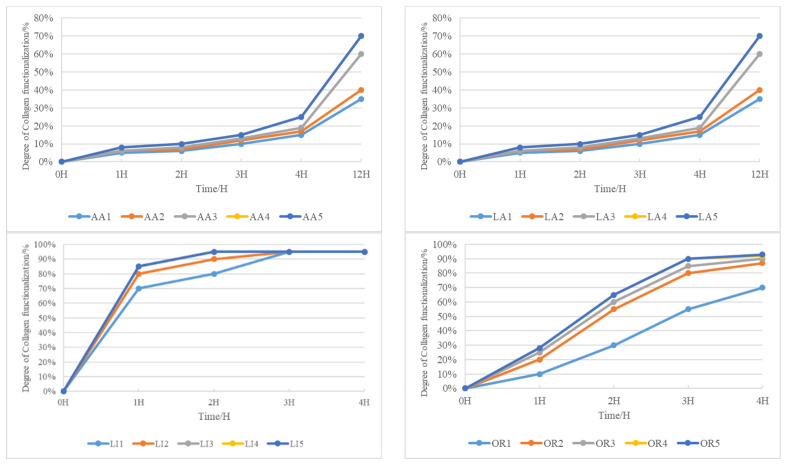
Study of the solubilization of collagen by different products.

After preparing the different solutions using collagen and acetic acid (AA), lactic acid (LA), lime (LL), and trypsin (OR), the effectiveness of each product was studied. See Figure 1.

The efficacy of collagen modification with acetic acid exhibits a gradual improvement, becoming more pronounced only after an overnight period. Interestingly, the impact of an increased acetic acid quantity is not immediately apparent within a short timeframe. It takes a prolonged exposure of 12 h for the concentration of acetic acid to significantly enhance collagen modification, reaching its peak at 70%. This underscores the necessity for an extended duration to achieve optimal results when employing acetic acid for collagen modification.

Similar observations apply to the use of lactic acid in collagen modification. The effectiveness is not immediately evident with a short exposure or increased lactic acid concentration. However, after an overnight period, a more conspicuous promotion effect becomes apparent. It takes 12 h for the concentration of lactic acid to significantly enhance collagen modification, reaching a maximum of 70%. Again, this emphasizes the requirement for an extended duration for optimal collagen modification when utilizing lactic acid.

Contrastingly, the modification of collagen with lime occurs rapidly and is completed in just two hours. However, the resulting collagen-based product exhibits a two-phase phenomenon, indicating severe peptide bond disruption due to the intense alkalinity of lime.

In the realm of enzyme-mediated modification, both enzyme quantity and time significantly influence collagen modification. Increasing enzyme quantity enhances modification effectiveness, reaching a saturation point at 2.0 g. Simultaneously, the extension of time greatly improves collagen modification, almost reaching saturation at 4 h. The deepening color of the solution with increased enzyme dosage is noted, and after 5 days, no two-phase phenomenon appears. This rapid and gentle functionalization of collagen using enzymes underscores its efficiency as a modification agent.

**Figure 2:**
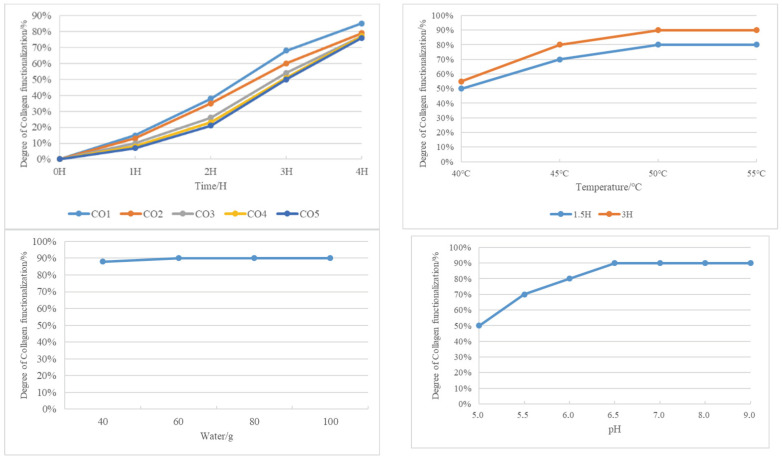
Relationship between different parameters and collagen solubilization.

After determining trypsin to be the most suitable modification agent, different parameters were studied (Figure 2): amount of collagen (CO), temperature, amount of water, amount of pH, and amount of trypsin.

Increasing the amount of collagen inversely affects its functionalization. At collagen amounts ranging from 5.0 g to 7.0 g, there is minimal difference in solubility after 4 h of functionalization. Opting for 7.0 g of collagen is deemed most suitable, taking into account the desired solid content of the final collagen-based product.

Elevated temperatures expedite collagen modification. The accelerated process is particularly noticeable with enzyme- and water-driven modifications. At 3 h, modification efficiency reaches 90%. Among the temperatures tested (40 °C, 45 °C, 50 °C, and 55 °C), 50 °C attains saturation, leading to the decision to set the modification temperature at 50 °C.

At 50 °C and 3 h, varying water amounts show a minimal impact on collagen functionalization, highlighting the predominant role of enzyme quantity. Setting the water amount at 40 g aligns with the desired solid content of the collagen-based coating product.

Two hours into the ultrasonic treatment, collagen functionalization is nearly complete. However, at pH levels 8 and 9, solution stratification occurs. The experiment halts, and subsequent analysis suggests that, despite swift functionalization, certain pH conditions result in undesired outcomes.

Collagen functionalization speed increases with rising pH levels, reaching completion in 2 h. Optimal functionalization is achieved at a pH of 6, with no further improvement. Beyond a pH of 8, a two-phase appearance suggests overfunctionalization. Considering future use in anionic and cationic finishing materials, a neutral pH of 7 is deemed optimal.

**Figure 3:**
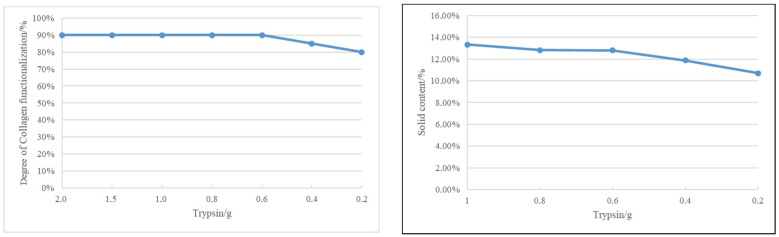

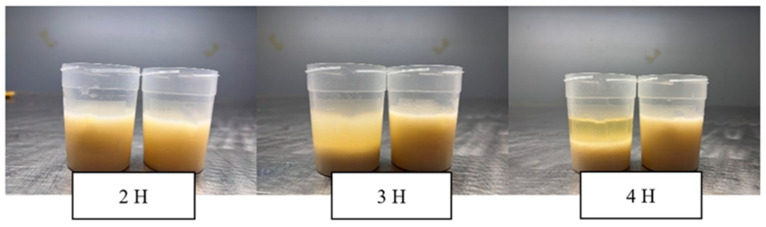
Study of the optimum conditions for collagen solubilization.

In order to obtain the optimum collage gel product, under the same conditions, T = 50 °C, t = 2 h, and pH = 7.0, the amount of trypsin and time were studied. See Figure 3.

From the depicted graph, it is evident that, for consistent collagen, temperature, time, and pH conditions, the trend in collagen functionalization leans toward a decrease as the enzyme amount diminishes. Though the differences are not substantial, we pragmatically opted for the enzyme quantities of 0.4 g and 0.2 g to delve into the nuanced interplay among collagen functionalization, time, and cost considerations associated with enzyme usage.

Examining Figure 3, it becomes apparent that at 0.4 g of trypsin, the collagen solution undergoes a two-phase transformation at the 3 h mark. This signifies that an elevated enzyme quantity, coupled with extended time, leads to an excess functionalization of collagen, resulting in the observed two-phase phenomenon.

Contrastingly, when employing a trypsin quantity of 0.2 g, our collagen-based finishing products exhibit no two-phase phenomenon. This suggests that the enzyme dosage of 0.2 g aligns more suitably with the desired outcomes.

At the 0.2 g trypsin level, collagen functionalization sees an increase with time, almost reaching saturation by the 3 h mark. Consequently, we propose that the optimal enzyme quantity for our purposes is 0.2 g, and the most effective time for collagen modification is 3 h.

The culmination of these findings is encapsulated in Table 1, presenting the final formulation.

The final collagen gel product was characterized as shown in Table 2.

**Figure 4:**
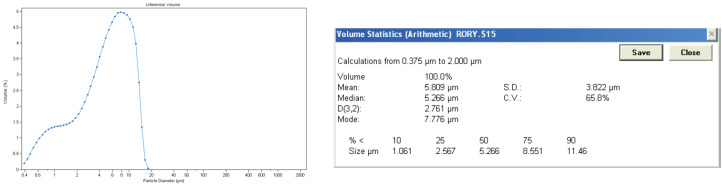
Particle size of collagen gel product by Coulter counter.

The particle size was measured by Coulter counter as can be seen in Figure 4.

The particle size of the collagen gel product is not homogeneous, and it has a wide range from 0.3 µm to 20 µm. As it comes from bovine waste, which is a natural material, it is difficult to obtain a homogeneous solution. Its average particle size is 5.809 µm, and 75% of the particles in the collagen gel product have a particle size below 8.551 µm, which indicates that they tend to stay on the surface rather than penetrate deeply into the material.

**Figure 5:**
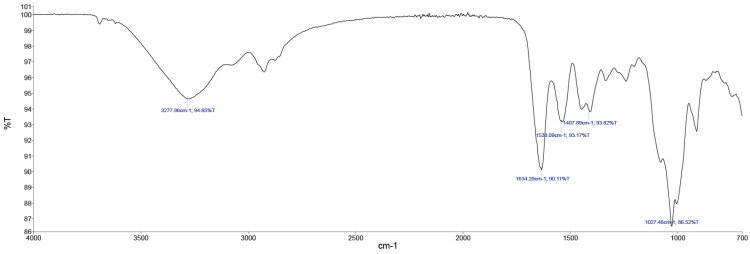
FTIR spectrum of collagen gel product.

In Figure 5, the FTIR spectroscopy of the collagen gel product is evident.

The main FTIR peaks include amide A (3277 cm^−1^), amide B (2919 cm^−1^), amide I (1634 cm^−1^), amide II (1538 cm^−1^), and amide III (1234 cm^−1^) as shown in Figure 5. The peaks were matched well with those of cow hide. Overall, the FTIR confirmed that the trypsin hydrolysis did not affect the triple-helical structure of collagen.

### 2.2. Leather Property Evaluation

Once optimized, the evaluations of the collagen gel products with different base coat formulations were carried out according to Table 3, Table 4, Table 5, Table 6 and Table 7. BC control is a conventional formulation, and BC 1, BC 2, and BC 3 are the collagen gel product substitutes of some of the conventional polymers used in the base coat. The physical properties obtained by using different formulations can be seen in Table 3.

According to the findings in Table 3, the collagen gel product exhibits a noteworthy enhancement in the water vapor permeability of the coating. This improvement contributes to an enhanced level of comfort of the final leather good.

In terms of color fastness to artificial light, the collagen gel product demonstrates remarkable stability, as there is no observed change in color fastness to artificial light when applied to the coating. This underscores the product’s high resistance to fading under artificial light conditions.

All samples showcase commendable color fastness to dry rubbing. However, when evaluating color fastness to wet rubbing and color fastness to water spotting, an interesting trend emerges. As the percentage of collagen gel product in the samples increases, there is a reduction in color fastness to wet rubbing. This suggests that while the product exhibits excellent color fastness to dry rubbing, there is room for improvement in its performance under wet rubbing conditions. This phenomenon aligns with the inherent characteristics of protein binders.

In a parallel way, different top coat formulations were tested on the same base coat formulation (BC control). The physical properties obtained by using different formulations can be seen in Table 4.

Upon application of the collagen gel product to the top coat, all test results yielded remarkably high values. This implies that its performance is comparable to that of conventional polymers when utilized in the top coat. However, a nuanced observation arises when considering the tactile experience. A subtle increase in stickiness is discernible on the top coat treated with the collagen gel product. This heightened stickiness indicates a pronounced water absorption capacity, resulting in a tendency to absorb moisture on the top coat and consequently imparting a slightly sticky texture upon touch.

Finally, different proportions of collagen gel products were tested to obtain a full biobased coating. The physical properties obtained are shown in Table 5.

The table reveals that Sample 8 outperforms the conventional coating. Moreover, the complete biobased coating (Sample 9) exhibits excellent performance across various parameters, with the exception of wet resistance. Notably, it excels in both touch and appearance. This confirmation underscores the applicability of the new collagen gel product for finishing bovine hides, meeting all the stringent requirements of the leather market.

## 3. Conclusions

The study highlights the potential of a collagen gel product as a sustainable and eco-friendly alternative for leather finishing. After exploring different proportions of the collagen gel product, the optimum product contains 5% collagen and 0.03% trypsin. The product exhibits a wide particle size range and retains the triple-helical structure of collagen. The base coat and top coat formulations using the collagen gel product were tested for various physical properties, such as water vapor permeability, flex resistance, color fastness, and adhesion. The results show that the collagen gel product enhances water vapor permeability and color stability in the finishes. In addition, it shows excellent performance in terms of touch and appearance, outperforming conventional coatings. However, a low resistance to wet rubbing is obtained; therefore, it is necessary to study how to improve this parameter.

The collagen-based product demonstrated promising results and showed potential for widespread application in the leather industry. However, further research and optimization are necessary to fine-tune the formulation and application techniques for large-scale production.

## 4. Materials and Methods

This section covers experimental procedures used during the obtainment of the new collagen gel product. The collagen-based product used in this study was developed through a controlled extraction and purification process from animal-derived byproducts. Different concentrations and formulations of collagen were prepared for testing.

There are previous studies on collagen extraction based on acidic, enzymatic, alkaline, and hot water extraction. The acid extraction method uses acidic conditions with low ionic concentration to break the gum salt and Schiff bonds between the original molecules and the ammonia bonds between different protein molecules, causing the collagen to swell and dissolve, so that the uncross-linked collagen is functionalized in the solution [28]. Hydrochloric acid, acetic acid, citric acid, and methyl acid are common preparative media, among which the most common and effective medium is acetic acid [29].

The enzymatic extraction method uses proteases to restrict the enzymatic digestion of collagen. The lysine or hydroxylysine at the end of the collagen molecule interacts to form covalent cross-linking bonds between collagen peptide chains and peptides. The collagen is still interconnected, and the collagen can be functionalized in low concentrations of organic acids or neutral solutions [30]. Due to the fact that proteases are specific and efficient, the enzyme method is the most popular method of collagen production. The most-used enzymes include pepsin, trypsin, neutral protease, pineapple protease, and papain [31]. Pepsin is the most commonly used enzyme. It is characterized by fast hydrolysis, high collagen purity, good solubility, and stable physicochemical properties, but it needs to be produced at a low temperature, which will reduce the activity of pepsin. Pepsin only catalyzes the hydrolysis of the nonhelical region of collagen and has no effect on the helical region, so the collagen produced will retain its intact triple-helix structure, but its antigenicity will be reduced; hence, it is commonly used as a medical biomaterial.

The alkaline extraction method consists of the extraction of collagen through the use of different alkaline products, such as lime, sodium hydroxide, and sodium carbonate. Sodium hydroxide is the most effective product. Because alkaline extraction tends to cause peptide bond hydrolysis, the molecular weight of the hydrolysis product obtained is low. Its disadvantages are strong reaction, strong collagen hydrolysis, and low nutrient content.

Finally, the hot water extraction method consists in increasing the dissolution rate of collagen by heating the material with hot water after pretreatment. When the hot water extraction method is used to extract collagen, the temperature and time should be controlled. This method has a long extraction time and low efficiency, and collagen is easily hydrolyzed into gelatine.

In this work, the alkaline method with lime was used to obtain collagen from bovine hide waste with the aim to study the optimum method to obtain a functionalized gel product suitable for biodegradable leather finishing. This study was performed in two steps. In the first one, the type of chemical agent, the temperature, the pH, and the amount of optimum chemical agent were studied. In the second step, different finishing formulations were studied in order to obtain an ecofriendly alternative for leather finishing.

### 4.1. Collagen-Based Product Development

To obtain the functionalized collagen, several solutions using collagen, distilled water, and different chemical agents were accurately weighed, put into beakers, and put into the ultrasonic machine to modify the collagen. The process of using ultrasound to solubilize collagen involves breaking down the collagen fibrils into smaller particles and dispersing them in a solvent. This method is commonly used to extract collagen from tissues or to prepare collagen solutions for various applications. Here is a general outline of the steps involved in performing the solution of collagen using ultrasound:

A solvent solution that contains the chemical agent (i.e., acetic acid, lactic acid, lime, and trypsin) was prepared. The chemical agent helps to solubilize collagen by disrupting the fibrils. A temperature of 50 °C was maintained throughout the process. The ultrasound waves will help break down the collagen fibrils further. Ultrasound was applied at a low intensity and frequency. We avoided using high power, as it might cause excessive heating and denaturation of the collagen. The use of ultrasound helps accelerate the breakdown of collagen fibrils and improves solubilization, but it should be carefully controlled to avoid damaging the collagen or causing denaturation.

Firstly, 20 solutions were prepared in order to select the most suitable chemical agent (see Table 6). Once the chemical agent was chosen, 33 more solutions were prepared to study the different variables, i.e., temperature, pH, amount of distilled water, and amount of chemical agent as can be seen in Table 7.

For the characterization of the optimal collagen-based product, the following techniques were performed:

- The pH was measured using a pH meter, according to ISO 4045:2018.
- The density was measured by the method UNE-EN 27888: 1994.
- The viscosity was measured by using Ford Cup Number 4.
- The particle size was measured by Coulter counter. A typical Coulter counter has one or more microchannels that separate two chambers containing electrolyte solutions. As fluid that contains particles or cells is drawn through the microchannels, each particle causes a brief change to the electrical resistance of the liquid. The counter detects these changes in the electrical resistance.
- Fourier-transform infrared spectroscopy was used to determine the functional groups of the resulting collagen-based coating product.

### 4.2. Application of Collagen Gel Product

Leather samples of uniform thickness and quality were selected for the experiments. The samples were sourced from a local tannery. The collagen gel product was applied to the leather samples using spraying. The treated samples were compared against control samples that received traditional chemical finishes.

The finishing formulations are shown in Table 8, Table 9, Table 10, Table 11 and Table 12. Table 8 and Table 9 are formulations for conventional anionic finishes. Table 10 and Table 11 are formulations for special finishes. And Table 12 is a formulation for biodegradable finishes.

Leather samples (25 cm × 20 cm) were sprayed twice with the base coat formulations using a spray gun, dried at 50 °C in a heater, and then pressed at 80 °C at 80 kg/cm^2^ for 2 s. The base coat was applied and dried again twice. Leather samples were then sprayed twice with the top coat formulations, dried at 50 °C in a heater, and then pressed at 80 °C at 80 kg/cm^2^ for 2 s. Finished leather was stored for 5 days to let the finishing settle. The proportions of the compounds were calculated according to the solid content.

To evaluate the performance of the new collagen gel product as a finishing agent, the following tests were performed:

- Water vapor permeability according to standard ISO 14268:2012—Determination of water vapor permeability.
- Resistance to continuous flexing according to standard ISO 5402-1:2017 Leather—Determination of flex resistance.
- Color fastness to artificial light, according to standard ISO 105-B02:2014 Textiles—Tests for color fastness.
- Color fastness in response to water spotting according to standard ISO 15700:1998 Leather—Tests for color fastness to water spotting.
- Adhesion of the finishing according to standard ISO 11644:2009 Leather—Test for adhesion of finish.

## Figures and Tables

**Table 1:** Collagen gel product formulation.

T = 50 °C	pH = 7.0	t = 3 H
Component		Weight/g
Collagen		7
Trypsin		0.2
Water		40

**Table 2:** Characterization of collagen gel product.

pH	6.9
Viscosity	18 s (Ford Cup #4)
Density	1.029 g/cm^3^
Solid content	13.40%
Aspect of the film	Glossy/Semitransparent

**Table 3:** Characterization of different base coats.

Samples Parameters	1	2	3	4
	BC (Control)	BC 1	BC 2	BC 3
Touch	Sticky	Smooth	Smooth	Smooth
Gloss	Shiny	Shiny	Shiny	Shiny
Determination of water vapor (mg/h·cm^2^)	4.3	11.2	8.4	10.2
Bally Flex 100,000 times (Dry)	Small wrinkles	Small wrinkles	Small wrinkles	Small wrinkles
Bally Flex 50,000 times (Wet)	Wrinkles	Wrinkles	Broken	Wrinkles
Color fastness to artificial light *	7	6–7	7	7
Color fastness to water spotting *	4–5	1	2–3	1
Color fastness to rubbing (Dry) *	4–5	4–5	5	3–4
Color fastness to rubbing (Wet) *	3–4	1–2	2–3	1–2
Adhesion of finishing (Dry)/N/cm	16.2	22.5	23.5	21.8
Adhesion of finishing (Wet)/N/cm	10.2	6.8	7.3	4.7

* A value of 1 indicates very poor fastness, and a value of 5 indicates very good fastness.

**Table 4:** Characterization of different top coats.

Samples Parameters	5	6	7	8
	BC + TC (Control)	BC + TC 1	BC + TC 2	BC + TC 3
Touch	Smooth	Semisticky	Semisticky	Semisticky
Gloss	Natural	Shiny	Shiny	Shiny
Determination of water vapor (mg/h·cm^2^)	4.3	4.2	3.6	4.5
Bally Flex 100,000 times (Dry)	Small wrinkles	Small wrinkles	Wrinkles	Wrinkles
Bally Flex 50,000 times (Wet)	Wrinkles	Broken	Wrinkles	Broken
Color fastness to artificial light *	7	7	7	7
Color fastness to water spotting *	4	4–5	4–5	4–5
Color fastness to rubbing (Dry) *	5	5	5	5
Color fastness to rubbing (Wet) *	4–5	4	4	4
Adhesion of finishing (Dry)/N/cm	9.7	13.3	11.5	16.4
Adhesion of finishing (Wet)/N/cm	4.3	2.6	5.5	3.9

* A value of 1 indicates very poor fastness, and a value of 5 indicates very good fastness.

**Table 5:** Characterization of biobased coatings.

Samples Parameters	5	8	9
	BC + TC (Control)	BC 8 + TC 8	BC 9 + TC 9
Touch	Smooth	Dry	Smooth
Gloss	Natural	Matte	Shiny
Determination of water vapor (mg/h·cm^2^)	4.3	4.2	3.6
Bally Flex 100,000 times (Dry)	Small wrinkles	Wrinkles	Wrinkles
Bally Flex 50,000 times (Wet)	Wrinkles	Wrinkles	Wrinkles
Color fastness to artificial light *	7	6–7	6–7
Color fastness to water spotting *	4	4	1
Color fastness to rubbing (Dry) *	5	5	4–5
Color fastness to rubbing (Wet) *	4–5	4–5	2
Adhesion of finishing (Dry)/N/cm	9.7	9.2	7.8
Adhesion of finishing (Wet)/N/cm	4.3	5.6	5.6

* A value of 1 indicates very poor fastness, and a value of 5 indicates very good fastness.

**Table 6:** Solutions to choose the best chemical to functionalize the collagen.

AA 1.		AA 2		AA 3		AA 4		AA 5	
Component	W/g	Component	W/g	Component	W/g	Component	W/g	Component	W/g
Collagen	5	Collagen	5	Collagen	5	Collagen	5	Collagen	5
Acetic acid	1	Acetic acid	2	Acetic acid	3	Acetic acid	4	Acetic acid	5
Water	100	Water	100	Water	100	Water	100	Water	100
**LA 1.**		**LA 2**		**LA 3**		**LA 4**		**LA 5**	
Collagen	5	Collagen	5	Collagen	5	Collagen	5	Collagen	5
Lactic acid	1	Lactic acid	2	Lactic acid	3	Lactic acid	4	Lactic acid	5
Water	100	Water	100	Water	100	Water	100	Water	100
**LI 1.**		**LI 2**		**LI 3**		**LI 4**		**LI 5**	
Collagen	5	Collagen	5	Collagen	5	Collagen	5	Collagen	5
Lime	1	Lime	2	Lime	3	Lime	4	Lime	5
Water	100	Water	100	Water	100	Water	100	Water	100
**TR 1.**		**TR 2**		**TR 3**		**TR 4**		**TR 5**	
Collagen	5	Collagen	5	Collagen	5	Collagen	5	Collagen	5
Trypsin	1	Trypsin	2	Trypsin	3	Trypsin	4	Trypsin	5
Water	100	Water	100	Water	100	Water	100	Water	100

**Table 7:** Study of different variables.

CO 1				CO 2				CO 3			CO 4					CO 5			
Component		W/g		Component		W/g		Component		W/g	Component			W/g		Component			W/g
Collagen		5		Collagen		5.5		Collagen		6	Collagen			6.5		Collagen			7
Trypsin		2		Trypsin		2		Trypsin		2	Trypsin			2		Trypsin			2
Water		100		Water		100		Water		100	Water			100		Water			100
**T 1 = 40 °C**					**T 2 = 45 °C**				**T 3 = 50 °C**						**T 4 = 55 °C**				
Component	Weight/g				Component		Weight/g		Component			Weight/g			Component			Weight/g	
Collagen	7				Collagen		7		Collagen			7			Collagen			7	
Trypsin	2				Trypsin		2		Trypsin			2			Trypsin			2	
Water	100				Water		100		Water			100			Water			100	
**WA 1**					**WA 2**				**WA 3**						**WA 4**				
Component	Weight/g				Component		Weight/g		Component			Weight/g			Component			Weight/g	
Collagen	7				Collagen		7		Collagen			7			Collagen			7	
Trypsin	2				Trypsin		2		Trypsin			2			Trypsin			2	
Water	40				Water		60		Water			80			Water			100	
**PH 1 = 5.0**					**PH 2 = 5.5**				**PH 3 = 6.0**						**PH 4 = 6.5**				
Component	Weight/g				Component		Weight/g		Component			Weight/g			Component			Weight/g	
Collagen	7				Collagen		7		Collagen			7			Collagen			7	
Trypsin	2				Trypsin		2		Trypsin			2			Trypsin			2	
Water	40				Water		40		Water			40			Water			40	
**PH 5 = 7.0**						**PH 6 = 8.0**							**PH 7 = 9.0**						
Component			Weight/g			Component			Weight/g				Component				Weight/g		
Collagen			7			Collagen			7				Collagen				7		
Trypsin			2			Trypsin			2				Trypsin				2		
Water			40			Water			40				Water				40		

**Table 8:** Base coat formulations for conventional anionic finishes.

Base Coat							
BC (Control)		BC 1		BC 2		BC 3	
Component	%	Component	%	Component	%	Component	%
Water	28	Water	28	Water	28	Water	28
Isopropanol	2	IPA	2	IPA	2	IPA	2
Polyurethane	34	Collagen	100	Polyurethane	34	Collagen	146
Acrylic resin	18	Acrylic resin	18	Collagen	46	Wax	2
Wax	2	Wax	2	Wax	2	Pigment	16
Pigment	16	Pigment	16	Pigment	16		

**Table 9:** Top coat formulations for conventional anionic finishes.

Top Coat							
TC (Control)		TC 1		TC 2			
Component	%	Component	%	Component	%	Component	%
Water	50	Water	50	Water	50	Water	50
Polyurethane	17.5	Collagen	51.5	Polyurethane	17.5	Collagen	90.1
Urethane	30	Urethane	30	Collagen	38.6	Wax	15
Wax	15	Wax	15	Wax	15		

**Table 10:** Base coat formulations for special finishes.

Base Coat							
BC 4		BC 5		BC 6		BC 7	
Component	%	Component	%	Component	%	Component	%
Water	28	Water	50	Water	20	Water	20
Isopropanol	2	Casein	50	Cationic wax	50	Cationic wax	50
Casein	9	Collagen	20	Collagen	40	Collagen	40
Wax	2	Wax	5	Cationic urethane	15	Cationic polyurethane	15
Protein binder	9						
Collagen	50						

**Table 11:** Top coat formulations for special finishes.

Top Coat							
TC 4		TC 5		TC 6		TC 7	
Component	%	Component	%	Component	%	Component	%
Water	28	Water	50	Water	20	Water	20
Isopropanol	2	Casein	50	Cationic wax	50	Cationic wax	50
Casein	9	Collagen	20	Collagen	40	Collagen	40
Wax	2	Wax	5	Cationic urethane	15	Cationic polyurethane	15
Protein binder	9						
Collagen	50						

**Table 12:** Formulations for biodegradable finishes.

Biodegradable Formulation			
BC 8		BC 9	
Water	35	Water	35
Isopropanol	2	Isopropanol	2
Biodegradable polyurethane	12.5	Collagen	72
Biodegradable urethane	10	Wax	2
Wax	2	Pigment	16
Pigment	16		

## Data Availability

The data presented in this study are openly available in article.
